# Reprogramming with Small Molecules instead of Exogenous Transcription Factors

**DOI:** 10.1155/2015/794632

**Published:** 2015-04-01

**Authors:** Tongxiang Lin, Shouhai Wu

**Affiliations:** ^1^Guangzhou University of Chinese Medicine, The Second Affiliated Hospital (Guangdong Provincial Hospital of Chinese Medicine), 55 Neihuan W. Road, Higher Education Mega Center, Guangzhou, Guangdong 510006, China; ^2^Fujian Agriculture and Forestry University, Stem Cell Research Center, 15 Shangxiadian Road, Cangshan District, Fuzhou, Fujian 350002, China

## Abstract

Induced pluripotent stem cells (iPSCs) could be employed in the creation of patient-specific stem cells, which could subsequently be used in various basic and clinical applications. However, current iPSC methodologies present significant hidden risks with respect to genetic mutations and abnormal expression which are a barrier in realizing the full potential of iPSCs. A chemical approach is thought to be a promising strategy for safety and efficiency of iPSC generation. Many small molecules have been identified that can be used in place of exogenous transcription factors and significantly improve iPSC reprogramming efficiency and quality. Recent studies have shown that the use of small molecules results in the generation of chemically induced pluripotent stem cells from mouse embryonic fibroblast cells. These studies might lead to new areas of stem cell research and medical applications, not only human iPSC by chemicals alone, but also safe generation of somatic stem cells for cell based clinical trials and other researches. In this paper, we have reviewed the recent advances in small molecule approaches for the generation of iPSCs.

## 1. Introduction

Human induced pluripotent stem cells (iPSCs) are similar to human embryonic stem cells in that they have the potential to differentiate into cells of all three germ layers [1–3]. Elderly patients with injuries, degenerative diseases, or cancers would benefit from stem cell-based regenerative medical techniques. The iPSC applications promise in cell transplantation and stimulate the regenerative medicine of endogenous cells to rebuild tissues,* in vitro* drug screening, and disease modeling.

Initially, adult cells were induced into iPSCs through exogenous overexpression of the transcription factors Oct4 (also known as Pou5f1), Sox2, cMyc, and Klf4. However, efficiency of this technique is at very low level, with around 0.1% of mouse fibroblasts [4] and 0.01% of human fibroblasts cell [2, 5]. The low efficiency and slow dynamics of this method posed serious potential problems for the generation of iPSCs. Besides low iPSC generation efficiency, there are some safety concerns regarding the overexpression of the four aforementioned transcription factors involving genetic mutations, gene insertions, epigenetic changes, incomplete reprogramming, and immunogenicity [6–12].

To improve the efficiency and quality of iPSC induction, much effort has been applied in the development of new iPSC generation methods through the use of integrating and nonintegrating recombinant viruses [13–18], DNA expression vectors [19], episomal vectors [20, 21], minicircle vectors [22], and liposomal magnetofection [23]. Non-DNA methods involving proteins [24, 25], mRNA molecules [26], and various chemical agents [27] have also been trialed, and a chemical method that produces chemically induced pluripotent stem cells (CiPSCs) appears to be the most promising methods [27].

Although the human iPSC by using chemicals only has not been developed yet, human stem cells studied with small molecules are revealing further details about epigenetic remodeling. Thus, hopefully these researches might relieve concerns about the specificity, efficiency, kinetics, and safety of generating human iPSCs and bring human iPSC closer to effective clinical use [28–30]. We here discuss the small molecules in iPSC generation including three types of compounds: small molecules that may improve reprogramming efficiency; compounds that replace one or more reprogramming factors; compound combination alone that is sufficient to induce mouse iPSC. We also provide perspective views of the possibility of the iPSC generation from human somatic cells and its future applications.

## 2. Compounds That May Improve the Reprogramming Efficiency and Quality

**Figure 1:**
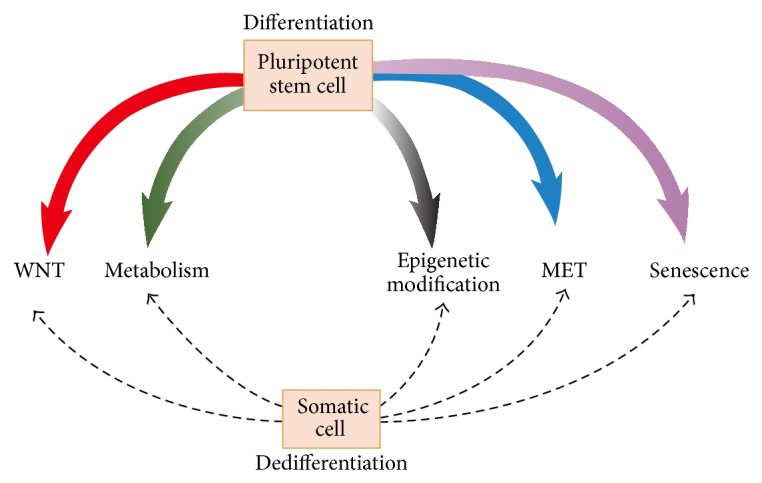
Differentiation and reprogramming are influenced by various mechanisms. Small molecules used to generate iPSCs can be categorized as epigenetic modifiers, WNT signal modulators, cell senescence moderators, modulators of metabolism, and regulators of MET and cell death/senescence pathways. They can influence both differentiation and reprogramming (dedifferentiation).

For the first time, Huangfu et al. studied the compound application in iPSC generation; they investigated the effects of the histone deacetylase (HDAC) inhibitor valproic acid (VPA) and found that reprogramming efficiency was increased 100-fold over that of the transcription factor method [31]. Soon, Ding group used BIX-01294, which inhibits histone methyltransferase (HMT) by activating calcium channels in the plasma membrane, to improve reprogramming efficiency [32, 33]. To date, the small molecules that have been used to generate iPSCs can be categorized as epigenetic modifiers, wingless and integration site growth factor (WNT) signal modulators, moderators of cell senescence, or modulators of metabolism [3, 34, 35] and the functions of the small molecules in iPSC generation are summarized in Figure 1. Through these mechanisms, the small molecules could improve iPSC generation efficiency and/or could replace some of Yamanaka factors. The small molecules that might enhance iPSC generation efficiency were collected in Table 1.

### 2.1. Epigenetic Modification

During programming, cells undergo changes at a global transcriptional level and also experience epigenetic changes in their DNA, along with histone modifications [3, 28, 29, 34, 36, 37]. Small molecules, such as Bix-01294 (Bix), target enzymes responsible for histone methylation and demethylation and increase expression levels of OCT4 and KLF4 during somatic cell reprogramming [32]. Parnate, a lysine-specific demethylase inhibitor (LSD1), mediates H3K4 demethylation, enabling Oct4 and KLF4 induction of human keratinocytes into iPSCs when combined with CHIR99021, a glycogen synthase kinase 3 (GSK-3) inhibitor [31]. DNA methyltransferase inhibitor 5-azacytidine (5-Aza) or three other histone deacetylase inhibitors (suberoylanilide hydroxamic acid, trichostatin A, and valproic acid) can also improve reprogramming efficiency following the transduction of MEFs with four transcription factors [31, 35, 38, 39].

To improve incomplete epigenetic reprogramming, the somatic memory can be erased and new epigenetics are established in iPSCs* via* treatment with trichostatin A [38, 39]. Epigenetic modification using small molecules significantly increases iPSC reprogramming efficiency and reduces epigenetic memory retention [3, 34, 39].

### 2.2. WNT Signal Modulators

The WNT pathway plays an important role in self-renewal of embryonic stem cells (ESCs) [40]. The CHIR99021 molecule, an inhibitor of GSK-3*β*, activates WNT signaling, improving the efficiency of programming and eliminating the use of c-Myc [41].

When combined with other compounds, they serve as replacements for certain key transcription factors, or they can induce differentiation into specific cell types. It has been hypothesized that WNT pathway regulators could play a significant role in cellular reprogramming. As an example, the GSK-3 beta inhibitor CHIR99021 can improve reprogramming efficacy by replacing c-Myc. Another GSK3 inhibitor, kenpaullone, can be used as a replacement for KLF4; however, the three transcription factors Oct4, Sox2, and c-Myc must still be used for successful reprogramming [42]. Many similar small molecules have been found to be effective in iPSC generation. The screening of other WNT signaling modulators has been discussed in detail by other researchers [34, 39, 40, 43].

### 2.3. Moderators of Cell Senescence

Cell senescence in reprogramming is usually thought to contribute to slow dynamics and low efficiency. Stress response, cell senescence, and early programming characteristics of iPSCs involve expression of p21cip1 and p16INK4a/p19arf, which are upregulated by p53. Knocking out p53 improves iPSC generation efficiency and kinetics [44–51]. However, the p53 protein is a key tumor suppressor and improving iPSC generation by blocking p53 likely increases the risk of tumor formation [44–50, 52], as shown in murine ESCs [51].

A common small molecule, natural antioxidant, vitamin C, has been found to promote the formation of iPSCs from mouse and human somatic cells by indirectly lowering the expression of p21 and p53 [53]. Some small molecules have been found to be regulators of the pathways involved in cell senescence, with little risk of tumor formation [54]. They downregulate the expression of various genes and result in improved efficiency and dynamics during iPSC generation [55–57].

### 2.4. Modulators of Metabolism

Growth of differentiated adult somatic cells usually involves mitochondrial oxidative phosphorylation, while pluripotent stem cells mainly employ glycolysis metabolism [56–58]. This metabolic programming ability is driven by hypoxia-inducible factor 1 alpha, with c-Myc promoting glycolysis [58]. Consistently, PS48, an activator of 3′-phosphoinositide-dependent protein kinase 1, in combination with other small molecules (a-83-01 and PD0325901) and sodium butyrate (a histone deacetylase inhibitor) has been used to reprogram adult keratinocytes, umbilical vein endothelial cells, and amniotic fluid-derived cells. It has been shown that PS48 activates the phosphoinositide 3-kinase/Akt pathway, upregulating gene expression which promotes mitochondrial oxidative phosphorylation and glycolysis metabolism [59].

### 2.5. MET Mediated by Transforming Growth Factor- (TGF-) *β* Pathway Signaling Inhibitors Enhances Reprogramming

A mesenchymal-epithelial transition (MET) is a reversible biological process involving the transition from motile multipolar or spindle-shaped mesenchymal cells to planar arrays of polarized epithelial cells. Reprogramming of fibroblast cells or iPSCs inevitably involves MET, with cells at various stages of reprogramming undergoing morphological changes towards an epithelial-like cell type [60, 61].

Several researchers have shown that inhibition of the TGF-*β* signaling pathway enhances reprogramming through derepressing the mesenchymal phenotype and inducing MET. By combining two small molecules, SB431542 (an inhibitor of the TGF-*β* receptor) and PD0325901 (a MEK inhibitor), we demonstrated a 100-fold improvement of efficiency of human iPSC generation [62].

Further features of MET were revealed in three other studies where small molecules were used to inhibit TGF-*β* signaling or E-cadherin upregulation. Among the many TGF-*β* inhibitors, E-616452 (also known as Repsox) was recently found to be a functional substitute for SOX2 in mouse fibroblast reprogramming with OKM; it also indirectly enhanced NANOG expression during the late stages of reprogramming [63]. The TGF-*β* receptor inhibitor, a-83-01, combined with the protein arginine methyltransferase inhibitor, AMI-5, along with OCT4, has also been found to promote reprogramming [64]. Small molecule modulators of signal pathways, alone or in combination, and sometimes with epigenetic modifications induced by exogenous transcription factors, affect reprogramming efficiency through the influence of an integrated cellular network.

## 3. Compounds That Can Replace Reprogramming Factors

To date, many studies on small molecules that could be used to replace reprogramming transcription factors have been published. Most of the small molecules might improve reprogramming efficiency and also could replace some functions of one or more Yamanaka factors, Oct4, Sox2, Klf4, c-Myc, or their combinations; we summarize the representative studies (Table 2).

### 3.1. Compounds That Can Replace Oct4 in Reprogramming

Oct4 is the master regulatory pluripotency gene and may serve as a pluripotency determinant in reprogramming. Several compounds have been claimed that might replace Oct4 expression (Table 2). BIX01294, a G9a HMTase inhibitor, was first reported to induce miPSCs in place of Oct4 [32]. RG108, a DNMT inhibitor, can replace Oct4 during mouse skeletal muscle cell reprogramming into miPSCs where skeletal muscle cells endogenously express Sox2, Klf4, and c-Myc [33].

Cellular reprogramming involves profound alterations in genome-wide gene expression that is precisely controlled by a hypothetical epigenetic code that can be created artificially by epigenetic modification in a sequence dependent manner with small molecules. A recent report claimed that a specific DNA binding hairpin pyrrole-imidazole polyamides (PIPs) could be conjugated with chromatin modifying histone deacetylase inhibitors like SAHA to epigenetically activate certain pluripotent genes in mouse fibroblasts and identified a novel compound termed SAHA-PIP delta [65]. It could dramatically induce the endogenous expression of Oct-3/4 and Nanog and other pluripotency associated genes; thereby the cells rapidly overcame the rate-limiting step of epithelial transition in cellular reprogramming by switching “ON” the complex transcriptional gene network [65].

However, Oct4 could also be replaced by other compounds, such as FSK under binodal “Seesaw” model (Hou et al. and Shu et al.). This mechanism is mentioned in detail in the section of the CiPSC. Many of the small molecules used to replace Oct4 might fall into this category.

### 3.2. Small Molecules That Might Replace Sox2

The compounds 616452 (E-616452, Repsox) and SB431542 are transforming growth factor- (TGF-) beta inhibitors that could replace Sox2 during mouse and human iPSC generation [63]. However, 616452 does not actually act by inducing Sox2 expression in the target cells; rather, it enables reprogramming through the induction of Nanog transcription as reported. Another TGF-b inhibitor, LY-364947, can replace Sox2 in miPSC generation [74].

BayK8644 (BayK), an L-channel calcium agonist, was also reported to be able to replace Sox2 in combination with BIX01294 during MEF reprogramming into miPSCs [32, 33, 72]. Shh, purmorphamine, and oxysterol, the activator of Sonic hedgehog signaling, have been reported to upregulate Bmi1, Sox2, and N-Myc expression in mouse fibroblasts [75].

Staerk et al. applied a cell-based, high-throughput chemical screening method to identify small molecules that can replace Sox2 during mouse somatic cell reprogramming [74]. From their Nanog reporter-based screening, they discovered that the Pan-Src family kinase inhibitors iPYrazine, dasatinib, and PP1 could replace Sox2 in MEF reprogramming into miPSCs [74].

### 3.3. Small Molecules That Might Replace Klf4 and c-Myc

While WNT signaling pathway regulators can improve iPSC generation efficiency, they also could replace the function of the c-Myc. Furthermore, several small molecules can increase the iPSC generation efficiency by replacing Klf4 and c-Myc during somatic cell reprogramming into iPSCs. Huangfu et al. reprogrammed MEFs into miPSCs using VPA and transducing three factors without introducing the oncogene c-My [31]. Kenpaullone has been reported as a substitute for Klf4 in mouse cells, although the underlying mechanism is unknown [42].

### 3.4. Small Molecules That Might Replace Sox2, Klf4, and c-Myc

Li et al. succeeded in generating miPSCs by transduction of Oct4 alone with the addition of VPA, CHIR99021, 616452, and tranylcypromine (VC6T) in the culture medium [73]. It is thought that the small-molecule combination including VC6T facilitated miPSC generation by lowering several major barriers to the reprogramming process. Really, the first iPSC generation protocol by using complete chemical alone is based on this protocol [27]. Similarly, many other small molecules combinations were also reported [29, 64, 66–68, 70–72, 75–79].

### 3.5. Combination of 2i Provides Mouse ESC Ground Status for Mature Mouse iPSC

As reported before, the combination of chir99021 (GSK-3 beta inhibitor) and PD0325901 (mitogen-activated protein kinase/extracellular signal-regulated kinase inhibitor), designated 2i, is required to maintain the pluripotent ground state in mouse ES cells [80]. Hou et al. cultured the partially reprogrammed cells in 2i medium with leukemia inhibitory factor which induced the stable upregulation of Oct4 and Nanog, transgene silencing, and competence for somatic and germline chimerism; thereby the cells were completely reprogrammed into mouse CiPSCs [27].

## 4. Compound Cocktails Required to Generate Chemical iPSCs (CiPSCs)

For the first time in history, Hou et al. obtained chemically induced pluripotent stem cells from mouse embryonic fibroblast cells after extensive screening compounds and compound cocktails (Table 3).

**Figure 2:**
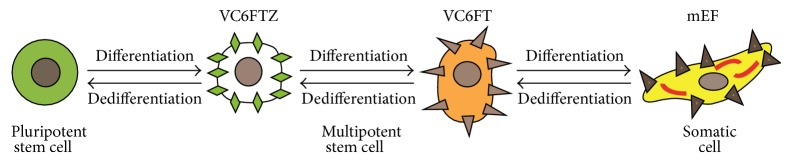
Small molecules based iPSC generation. Small molecules can substitute for all TFs through epigenetic modifications and signaling pathway regulations. Hou et al. reported that CiPSC generation from mEF was carried out in 3 steps of 16–20 days in VC6TF treatment and then 12–20 days in VC6TFZ and followed by 2i compounds regulations for 1 week. The somatic cell, mouse embryonic fibroblast (mEF) cell, undergoes dedifferentiation and gains multipotent stem cell characteristics under the treatments of VC6TF and VC6TFZ steps and finally obtains the full pluripotency in the medium containing 2i compounds (Chir99021 and PD0325901).

The CiPSC was induced through a very complicated procedure with 3-step compound treatment shown in Figure 2. To start, they searched for small molecules that enabled mEF reprogramming in the absence of Oct4 using mEF expressing Oct4 promoter-driven GFP to identify small molecules [27]. Three small molecules, Forskolin (FSK or F), 2-methyl-5-hydroxytryptamine, and D4476, were chemical substitutes for Oct4 after screening 10 000 compounds. FSK was then chosen for subsequent studies. Their early findings showed that the compounds cocktail “VC6T” (VPA, CHIR99021, 616452, and tranylcypromine) could induce mouse iPSC with a single-gene transduction of Oct4. Thus, they combined these two groups of small molecules into a cocktail, VC6TF, resulting in cells expressing E-cadherin [27]. However, Oct4 and NANOG expression were not detectable in these cells, suggesting that reprogramming was incomplete [27].

They found one more compound, 3-deazaneplanocin (DZNep or Z, epigenetic modulator), whose chemical induced stem cell medium contained VC6TFZ [27]. To enhance more pluripotent stem cell gene expression, they furthermore treated the cell in mouse pluripotent ground status conditions with ES medium containing LIF and PD0325901 and CHIR99021 (2i medium).

They designed CiPSC protocol including 3 steps as follows: (a) the mEF cells were cultured in mESC medium containing VC6FT for 16–20 days; (b) the cells were cultured in the medium with VC6FTZ for 12–20 days; (c) the cells were cultured in mESC medium containing 2i (PD0325901 and Chir99021) for 1 week. The characteristics of CiPSCs resembled mESCs in terms of their gene expression profiles, epigenetic status, and potential for differentiation and germline transmission. Therefore, they completed the CiPSC prepared from mEF solely using a combination of seven small-molecule compounds without using transduction or transfection of TFs [27].

The group also found that among seven compounds, individual small molecule of C, 6, F, Z was the most essential ingredient to CiPSC; removal of any one of these compounds from the cocktail might result in failure of CiPSC [27]. While these data are very interesting and clearly showed that CiPSC was generated from mEF, the principle of the CiPSC is not clear. To explore the mechanism of the small molecules based iPSC, the research group reported new findings on mechanisms of the pluripotent stem cell status [37].

For years, it was generally believed that ESCs are maintained by a shield of pluripotency factors which function in concert with each other to prevent ESCs from differentiating into any lineage, thus preserving ESCs at an undifferentiated state. Noting that some small molecules could replace the Yamanaka factors individually, the combination of these small molecules should be sufficient to replace the Yamanaka Factors. Unfortunately, while some compounds could induce Oct4 expression, the key iPSC transcription factors and the other Yamanaka factors could not be replaced by a single set of small molecules. Therefore, Deng group performed many studies to discover the chemical based iPSC generation mechanism; a binodal “Seesaw” model for cell fate determination then was discovered [37].

Shu et al. proposed a new model, termed the “Seesaw” model, in which the pluripotent state is a precarious balancing equilibrium that results from continuous mutual competition between rival lineage specification forces. While the reprogramming factors that induce pluripotency have been identified primarily from embryonic stem cell- (ESC-) enriched pluripotency-associated factors, pluripotency can be induced with lineage specifiers that suppress ESC identity using pluripotency rivals, most of which are not enriched in ESCs. They found that OCT4 and SOX2, the core regulators of pluripotency, can be replaced by lineage specifiers that are involved in mesendodermal (ME) and ectodermal (ECT) specification, respectively [37].

OCT4 and its substitutes attenuated the elevated expression of a group of ECT genes, whereas SOX2 and its substitutes curtailed a group of ME genes during reprogramming. Surprisingly, the two counteracting lineage specifiers can synergistically induce pluripotency in the absence of both OCT4 and SOX2 [37]. Therefore, they concluded that the principle of the mouse CiPSC is that the groups of counteracting lineage specifier compounds, VC6FTZ, which belong to 2 counteracting lineage specifiers, were combined to induce the CiPSC [37].

After mouse CiPSC, the next logical step is to try using the CiPSC approach to make human stem cells. This binodal model shed light on fundamental questions regarding the establishment of cellular identity during programming in mouse. However, there are a lot of differences between mouse pluripotent stem cells and human ones. For example, leukemia inhibitory factor, LIF, is a necessary factor for mouse pluripotent stem cell, whereas the bFGF is a key essential supplement for human pluripotent stem cell culture. The subtle “Seesaw” balances of the pluripotent factors might need to be adjusted to specific conditions suitable for human CiPSC.

## 5. Conclusions and Perspectives

### 5.1. Is the Combination of Small Molecule Compounds Alone Sufficient for Human iPSC Generation?

To date, small molecules based iPSC have brought dramatically changes to iPSC research. Some of these small molecules increase reprogramming efficiency and quality, while others or combinations of them replace iPSC factors. Mouse CiPSCs were obtained, and thus the complete small molecule-based reprogramming, in a directed and deterministic manner, has fundamentally changed the reprogramming paradigm through a mechanism that involves the activation of endogenous transcription factors by small molecules instead of exogenously expressed transcription factors. Is the combination of small molecule compounds alone sufficient for human iPSC generation?

### 5.2. Extensively Screening for Novel Oct4 Substitutes and Other ESC-Related Transcription Factors

To our knowledge, hiPSC generation with small molecule cocktails has not yet been reported. While most factors used as reprogramming transgenes can be replaced by other means, Oct4 remained essential until the development of human CiPSC technology. Furthermore, the Oct4 expression level in the mouse CiPSCs was reported much lower than those in the mouse ESCs. In human iPSC generation, activation of Oct4 expression and reaching a high enough level similar to that in human ESC are a key challenge. New specific small molecule targeted to activation of reprogramming factors, such as Oct4, might be useful strategy to solve this problem as recent report [65]. Extensively screening for small molecules and compound modifications for novel Oct4 substitutes and other ESC-related transcription factors will cast light on the discovery of molecular mechanisms for reprogramming and transdifferentiation, which in turn facilitate the development of safer and more efficient stem cell resource for clinical applications.

### 5.3. Mechanisms Governing Human CiPSC Generation Might Need to Be Discovered

Deng group found that the two counteracting lineage specifiers can synergistically induce pluripotency in the absence of both OCT4 and SOX2 [37]. While the Seesaw model might also work in human iPSC generation, human pluripotent stem cell ground status conditions might be very different from mouse ones. For example, leukemia inhibitory factor, LIF, is necessary factor for mouse pluripotent stem cell, whereas the bFGF is a key essential supplement for human pluripotent stem cell culture. As a result, similar “seesaw model” of the pluripotent factors needs to be confirmed in human iPSC, and different mechanisms that specifically govern the human CiPSC generation might need to be discovered.

In addition, complete reprogramming in mouse CiPSC need mouse ESC ground status compound combination of chir99021 and PD0325901, designated 2i, are required to maintain the pluripotent ground state [80] and confirmed it is necessary for mouse CiPSC. However, 2i is useful but not sufficient in maintaining the ground state for human ESCs, while the compound Chir99021 is useful for iPSC proliferation when it is used with three small molecules during two-step iPSC induction [81].

**Figure 3:**
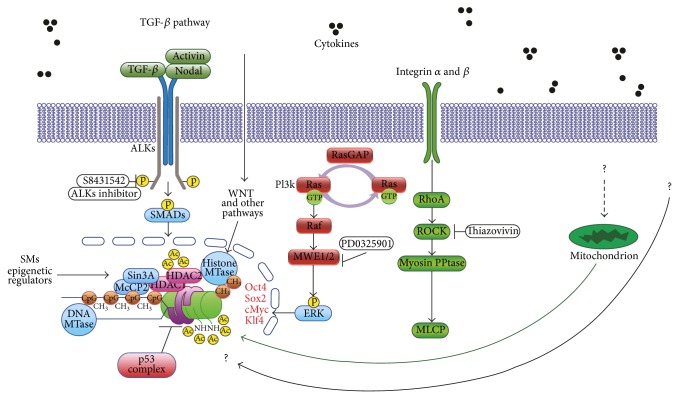
Human CiPSC perspective regulation pathways. The human CiPSC generation is so complicated that needs a lot of cellular reorganization, signaling pathways changes, and extracellular matrix maintaining conditions to achieve final reprogrammed pluripotent stem cell status. The cells were induced by chemicals through epigenetic modifications that switch on pluripotency transcription factors, such as Oct4, Nang, Sox2, Klf4, and c-Myc. The cell signaling pathways including TGF beta, WNT, ERK, ROCK, mitochondria, and other signaling pathways might need to be regulated in human CiPSC generation. However, waiting to be discovered mechanisms might also be some other unknown cell signaling pathways as well as nuclear and mitochondria genomic activities.

Furthermore, the human CiPSC generation is so complicated that it needs a lot of cellular reorganization, signaling pathways changes, and extracellular matrix maintaining conditions to achieve final reprogrammed pluripotent stem cell status (Figure 3). The cells were induced by chemicals through epigenetic modifications that switch on pluripotency transcription factors, such as Oct4, Nang, Sox2, Klf4, and c-Myc, and were kept on through chromatin remodeling. Conversely, genes responsible for differentiation must be turned off by the transcription machinery and kept silent through epigenetic mechanisms. While critical chemicals for pluripotency were VC6TFZ (C6FZ were essential) in mouse CiPSC generation, we might have to regulate more cell signaling pathways including TGF beta, WNT, ERK, ROCK, mitochondria, and other signaling pathways as found in human iPSC generation through viral or nonviral transfection till now. Therefore, waiting to be discovered mechanisms might also be a lot more, including cell signaling pathways as well as nuclear and mitochondria genomic activities (Figure 3).

### 5.4. Safety Issues, Especially, Tumor Concern, Have to Be Solved before Clinical Applications

Although human iPSCs provide an alternative stem cell resource for regenerative medicine, we still hesitate before using them in clinic applications due to safety concerns, such as tumor risks. The iPSC generation is so complicated that cellular reorganizations, signaling pathways changes, and extracellular matrix maintain conditions to achieve final reprogramming goals. Furthermore, it is extremely demanding to obtain highly pure population of the target cells. The small molecules based approaches might provide solutions for pure target adult stem cell without tumor concerns.

### 5.5. The Small Molecules Research Might Bring Close Future Applications Not Only for iPSC Application but Also for Adult Stem Cell Based Applications

The small molecules in the iPSC generation might also provide useful information for those studies in adult stem cell. Direct reprogramming of one type of adult stem cell to another one was suggested as an attractive alternative for clinical applications. Some studies have developed small molecules that guide human transdifferentiation of tissue-specific progenitor cells or stem cells. Because adult stem cells, such as bone marrow stem cells and hematopoietic stem cells, are ready to be used in clinical applications, the only obstacle is their limited proliferation. The development of small molecules to work in adult stem cell self-renewals or to guide human somatic cells into progenitor cells will open avenues for the clinical application of these types of progenitor cells and stem cells.

Advances in the understanding of reprogramming mechanism and continuous development of small molecular tools to enhance reprogramming will not only facilitate the possibility of generating safer and higher quality reprogrammed cells but also provide useful information for adult stem cell based applications.

## Figures and Tables

**Table 1:** Small molecules that enhance iPSC generation efficiency and quality.

Target or signaling pathway	Name	Concentration	Host cells	Efficiency and necessity	Reference
HDAC inhibitor	VPA	0.5–2 mM	Mouse, human	*>100-fold *	Huangfu et al. (2008) [31]
HDAC inhibitor	SAHA	5 *μ*M	Mouse	*10-fold *	Huangfu et al. (2008) [31]
HDAC inhibitor	TSA	20 nM	Mouse	*10-fold *	Huangfu et al. (2008) [31]
HDAC inhibitor	Sodium butyrate	0.5–1 mM	Mouse, human	*100-fold *	Mali et al. (2010) [66]
DMNT inhibitor	5-aza-CR, AZA	0.5 mM	Mouse	*3-fold *	Mikkelsen et al. (2008) [29]
DMNT inhibitor, histone deacetylase inhibitor	RSC133	10 *μ*M	Human	*3-fold *	Lee et al. (2012) [10, 67]
Retinoic acid receptor agonist	AM580	100 nM	Mouse	~200-fold	Wang et al. (2011) [68]
H3K4 demethylation inhibitor (epigenetic modulator)	Tranylcypromine (Parnate)	5–10 *μ*M	Mouse	*3-fold *	Li et al. (2009) [35]
Epigenetic modulators	DZNep	0.05–0.1 *μ*M	Mouse	65-fold	Hou et al. (2013) [27]
Retinoic acid receptor ligand	TTNPB	1 *μ*M	Mouse	*15-fold *	Hou et al. (2013) [27]
ALK4, ALK5, and ALK7 inhibitor	SB431542	10 uM	Human	Thiazovivin and PD0325901, ~200 fold	Lin et al. (2009) [62]
Selective MEK/ERK inhibitor	PD0325901	1 uM	Human	Thiazovivin and SB431542, ~200-fold	Lin et al. (2009) [62]
Rho-associated protein kinase inhibitor	Thiazovivin	1 uM	Human	PD0325901 and SB431542, ~200-fold	Lin et al. (2009) [62]
Rho-associated protein kinase inhibitor	Y27632	10 uM	Human	Improve generation and maintaining	Claassen et al. (2009) [69]
AKt-mediated inhibitor of GSK3-*β*	Compound B6	1 *μ*M	Mouse	*3-fold *	Li et al. (2009) [35]
GSK-3*β* inhibitor, LSD1 inhibitor	LiCl	5–10 mM	Mouse and human	*>10-fold *	Wang et al. (2011) [68]
TGF-*β* inhibitor	A83-01	0.5 *μ*M	Mouse, human	*7-fold *	Zhu et al. (2010) [59]
Prolyl-4-hydroxylase inhibitor	N-Oxalylglycine	1 *μ*M	Human		Zhu et al. (2010) [59]
ALK4 inhibitor	Compound B4 (TGFb-RI)	1 *μ*M	Mouse	4-fold	Li and Rana (2012) [70]
mTOR inhibitor	Rapamycin	0.3 nM	Mouse	4.8-fold	Chen et al. (2011) [71]
IP3K inhibitor	Compound B8	1-2 *μ*M	Mouse	*3-fold *	Li et al. (2009) [35]
P38 kinase inhibitor	Compound B10	1-2 *μ*M	Mouse	*3-fold *	Li and Rana (2012) [70]
cAMP agonist	Prostaglandin E2	5 *μ*M	Mouse	*Efficient in mixture *	Hou et al. (2013) [27]
cAMP agonist	Rolipram	10 *μ*M	Mouse	*Efficient in mixture *	Hou et al. (2013) [27]
cAMP-dependent protein kinase activator	8-Br-cAMP	0.1–0.5 mM	Human	6.5-fold	Wang et al. (2011) [68]
PDK1 activator	5-(4-Chloro-phenyl)-3-phenyl-pent-2-enoic acid (PS48)	5 *μ*M	Human	15-fold	Zhu et al. (2010) [59]
HIF PHD1 and PHD2 inhibitor	N-Oxalylglycine	1 uM	Human	3.5-fold	Zhu et al. (2010) [59]
Phosphofructokinase 1 activator	Fructose 2,6-bisphosphate	10 mM	Human	2-fold	Zhu et al. (2010) [59]
Hypoxia-inducible factor pathway activator	Quercetin	1 *μ*m	Human	3-fold	Zhu et al. (2010) [59]
Oxidative phosphorylation uncoupler	DNP	1 *μ*M	Human	2-fold	Zhu et al. (2010) [59]

Note: small molecules can improve reprogramming efficiency by epigenetic modifications or signaling pathway regulation. Many of these small molecules or compound combinations can also replace c-Myc or other transcription factors. DNP, 2,4-dinitrophenol; DZNep, 3-deazaneplanocin; FSK, forskolin; HDAC, histone deacetylase; IP3K, inositol triphosphate 3-kinase; PDK1, phosphoinositide-dependent kinase 1; SAHA, suberoylanilide hydroxamic acid; TF, transcription factor; TSA, trichostatin A; VPA, valproic acid; 2-Me-5HT, 2-methyl-5-hydroxytryptamine; 5-aza-CR, AZA, 5-azacytidine; 8-Br-cAMP, 8-Bromoadenosine cyclic monophosphate.

**Table 2:** Small molecule compounds that might replace Yamanaka factors.

Replacement for TF	Name concentration	Concentration	Host cell species	Function or target	Reference
Oct4, Nanog	SAHA-PIP delta	100 nM	Mouse	*Histone deacetylase inhibitor *	Pandian et al. (2014) [65]
Sox2 (with BIX) or Oct4	RG108	0.04–500 *μ*M	Mouse	DMNT inhibitor	Shi et al. (2008) [32, 33, 72]
Oct4	BIX	0.5–2 *μ*M	Mouse	G9a HMTase inhibitor	Shi et al. (2008) [32, 33, 72]
Sox2	CHIR	3–10 *μ*M	Mouse, human	GSK-3*β* inhibitor that activates Wnt signalling pathway	Li et al. (2011) [73]
Klf4	Kenpaullone	5 *μ*M	Mouse	GSK-3/CDKs inhibitor	Lyssiotis et al. (2009) [42]
Sox2	616452 (E-616452, Repsox)	1 *μ*M	Mouse, human	TGF-*β* inhibitor (ALK inhibitor II)	Ichida et al. (2009) [63]
Sox2	LY-364947	1 *μ*M	Mouse	TGF-*β* inhibitor	Staerk et al. (2011) [74]
Sox2, Klf4 (with A-83-01)	AMI-5	5 *μ*M	Mouse	Protein arginine methyltransferase inhibitor	Yuan et al. (2011) [64]
Sox2	Dasatinib	0.5 *μ*M	Mouse	Src family tyrosine kinase inhibitor	Staerk et al. (2011) [74]
Sox2	iPYrazine (iPY)	10 *μ*M	Mouse	Src family tyrosine kinase inhibitor	Staerk et al. (2011) [74]
Sox2	PP1	10 *μ*M	Mouse	Src family tyrosine kinase inhibitor	Staerk et al. (2011) [74]
Oct 4 with FSK and 2-Me-5HT	D4476	5 *μ*M	Mouse	CK1 inhibitor	Hou et al. (2013) [27]
Sox2	BayK	2 *μ*M	Mouse	An L-channel calcium agonist	Shi et al. (2008) [32, 33, 72]
Oct4, when used with 2-Me-5HT, and D4476	FSK	10–50 *μ*M	Mouse	cAMP agonist	Hou et al. (2013) [27]
Oct4 with FSK and D4476	2-Me-5HT	5 *μ*M	Mouse	5-HT3 agonist	Hou et al. (2013) [27]
Sox2, Klf4, and C-Myc	Oxysterol	0.5–1 *μ*M	Mouse	Sonic hedgehog signaling	Moon et al. (2011) [75]
Sox2, Klf4, and C-Myc	Purmorphamine	0.5–1 *μ*M	Mouse	Sonic hedgehog signaling	Moon et al. (2011) [75]
Sox2, Klf4, and C-Myc	Shh	500 ng/mL	Mouse	Sonic hedgehog signaling	Moon et al. (2011) [75]

Note: small molecules can substitute for certain TFs and/or improve reprogramming efficiency by epigenetic modifications or signaling pathway regulation. BayK, Bay K8644; BIX, BIX-01294; CHIR, CHIR99021; CK1, casein kinase 1; DNMT, DNA methyltransferase; DNP, 2,4-dinitrophenol; DZNep, 3-deazaneplanocin; FSK, forskolin; HDAC, histone deacetylase; G9a HMTase, G9a histone ethyltransferase; IP3K, inositol triphosphate 3-kinase; PDK1, phosphoinositide dependent kinase 1; SAHA, suberoylanilide hydroxamic acid; TF, transcription factor; TSA, trichostatin A; VPA, valproic acid; 2-Me-5HT, 2-methyl-5-hydroxytryptamine; 5-aza-CR, AZA, 5-azacytidine; 8-Br-cAMP, 8-bromoadenosine cyclic monophosphate.

**Table 3:** Small molecules (VC6TFZ and 2i) that are used in mouse CiPSC.

Target or signaling pathway	Name and concentration	TF to be replaced	Efficiency	Reference
TGF beta pathway ALK5 inhibitor	Repsox (616452) 5–10 uM	Sox2, Myc	Essential	Hou et al. (2013) [27] Ichida et al. (2009) [63]
PKA agonist	Forskolin 20–50 uM	Oct4 expression (with SKM) Klf2, klf4 expression	Essential	Hou et al. (2013) [27]
WNT pathway regulator, GSK3 beta inhibitor	Chir99021 10 uM	Sox2, Myc	Essential	Hou et al. (2013) [27]
Histone methylation modulator, lysine methyltransferase EZH2 inhibitor	DENep 50–100 nM	Increase reprogramming	Essential	Hou et al. (2013) [27]
Not specific	TTNPB 5 uM	Nuclease receptor signaling modulator	More efficient	Hou et al. (2013) [27]
Not specific	VPA 0.5 mM	Histone deacetylase inhibitor	More efficient	Hou et al. (2013) [27]
PD0325901: selective MEK/ERK inhibitor Chir99021: WNT pathway regulator, GSK3 beta inhibitor	2i: PD0325901 1 uM Chir99021 10 uM	Increase Oct4, Nanog, Sox2 expression	More mature	Hou et al. (2013) [27]

Small molecules can substitute for all TFs through epigenetic modifications and signaling pathway regulations. Hou et al. [27] reported that CiPSC generation from mEF was carried out in 3 steps of 16–20 days in VC6TF treatment and then 12–20 days in VC6TFZ and followed by 2i compounds regulations for 1 week. The abbreviations of the small molecules are shown in Tables 1 and 2.

## References

[1] Takahashi K., Tanabe K., Ohnuki M. (2007). Induction of pluripotent stem cells from adult human fibroblasts by defined factors. *Cell*.

[2] Yu J., Vodyanik M. A., Smuga-Otto K. (2007). Induced pluripotent stem cell lines derived from human somatic cells. *Science*.

[3] Chin M. H., Mason M. J., Xie W. (2009). Induced pluripotent stem cells and embryonic stem cells are distinguished by gene expression signatures. *Cell Stem Cell*.

[4] Takahashi K., Yamanaka S. (2006). Induction of pluripotent stem cells from mouse embryonic and adult fibroblast cultures by defined factors. *Cell*.

[5] Takahashi K., Okita K., Nakagawa M., Yamanaka S. (2007). Induction of pluripotent stem cells from fibroblast cultures. *Nature Protocols*.

[6] Okita K., Ichisaka T., Yamanaka S. (2007). Generation of germline-competent induced pluripotent stem cells. *Nature*.

[7] Ohi Y., Qin H., Hong C. (2011). Incomplete DNA methylation underlies a transcriptional memory of somatic cells in human iPS cells. *Nature Cell Biology*.

[8] Xu H., Yi B. A., Wu H. (2012). Highly efficient derivation of ventricular cardiomyocytes from induced pluripotent stem cells with a distinct epigenetic signature. *Cell Research*.

[9] Bar-Nur O., Russ H. A., Efrat S., Benvenisty N. (2011). Epigenetic memory and preferential lineage-specific differentiation in induced pluripotent stem cells derived from human pancreatic islet beta cells. *Cell Stem Cell*.

[10] Lee S. B., Seo D., Choi D. (2012). Contribution of hepatic lineage stage-specific donor memory to the differential potential of induced mouse pluripotent stem cells. *Stem Cells*.

[11] Lister R., Pelizzola M., Kida Y. S. (2011). Hotspots of aberrant epigenomic reprogramming in human induced pluripotent stem cells. *Nature*.

[12] Zhao T., Zhang Z.-N., Rong Z., Xu Y. (2011). Immunogenicity of induced pluripotent stem cells. *Nature*.

[13] Loh Y. H., Yang J. C., de los Angeles A. (2012). Excision of a viral reprogramming cassette by delivery of synthetic Cre mRNA. *Current Protocols in Stem Cell Biology*.

[14] Kaji K., Norrby K., Paca A., Mileikovsky M., Mohseni P., Woltjen K. (2009). Virus-free induction of pluripotency and subsequent excision of reprogramming factors. *Nature*.

[15] Woltjen K., Michael I. P., Mohseni P. (2009). *PiggyBac* transposition reprograms fibroblasts to induced pluripotent stem cells. *Nature*.

[16] Stadtfeld M., Nagaya M., Utikal J., Weir G., Hochedlinger K. (2008). Induced pluripotent stem cells generated without viral integration. *Science*.

[17] Zhou W., Freed C. R. (2009). Adenoviral gene delivery can reprogram human fibroblasts to induced pluripotent stem cells. *Stem Cells*.

[18] Fusaki N., Ban H., Nishiyama A., Saeki K., Hasegawa M. (2009). Efficient induction of transgene-free human pluripotent stem cells using a vector based on Sendai virus, an RNA virus that does not integrate into the host genome. *Proceedings of the Japan Academy Series B: Physical and Biological Sciences*.

[19] Okita K., Nakagawa M., Hyenjong H., Ichisaka T., Yamanaka S. (2008). Generation of mouse induced pluripotent stem cells without viral vectors. *Science*.

[20] Yu J., Hu K., Smuga-Otto K. (2009). Human induced pluripotent stem cells free of vector and transgene sequences. *Science*.

[21] Okita K., Matsumura Y., Sato Y. (2011). A more efficient method to generate integration-free human iPS cells. *Nature Methods*.

[22] Jia F., Wilson K. D., Sun N. (2010). A nonviral minicircle vector for deriving human iPS cells. *Nature Methods*.

[23] Park H. Y., Noh E. H., Chung H.-M., Kang M.-J., Kim E. Y., Park S. P. (2012). Efficient generation of virus-free iPS cells using liposomal magnetofection. *PLoS ONE*.

[24] Zhou H., Wu S., Joo J. Y. (2009). Generation of induced pluripotent stem cells using recombinant proteins. *Cell Stem Cell*.

[25] Kim D., Kim C.-H., Moon J.-I. (2009). Generation of human induced pluripotent stem cells by direct delivery of reprogramming proteins. *Cell Stem Cell*.

[26] Warren L., Manos P. D., Ahfeldt T. (2010). Highly efficient reprogramming to pluripotency and directed differentiation of human cells with synthetic modified mRNA. *Cell Stem Cell*.

[27] Hou P., Li Y., Zhang X. (2013). Pluripotent stem cells induced from mouse somatic cells by small-molecule compounds. *Science*.

[28] Maherali N., Sridharan R., Xie W. (2007). Directly reprogrammed fibroblasts show global epigenetic remodeling and widespread tissue contribution. *Cell Stem Cell*.

[29] Mikkelsen T. S., Hanna J., Zhang X. (2008). Dissecting direct reprogramming through integrative genomic analysis. *Nature*.

[30] Sridharan R., Tchieu J., Mason M. J. (2009). Role of the murine reprogramming factors in the induction of pluripotency. *Cell*.

[31] Huangfu D., Maehr R., Guo W. (2008). Induction of pluripotent stem cells by defined factors is greatly improved by small-molecule compounds. *Nature Biotechnology*.

[32] Shi Y., Desponts C., Do J. T., Hahm H. S., Schöler H. R., Ding S. (2008). Induction of pluripotent stem cells from mouse embryonic fibroblasts by Oct4 and Klf4 with small-molecule compounds. *Cell Stem Cell*.

[33] Shi Y., Do J. T., Desponts C., Hahm H. S., Schöler H. R., Ding S. (2008). A combined chemical and genetic approach for the generation of induced pluripotent stem cells. *Cell Stem Cell*.

[34] Nie B., Wang H., Laurent T., Ding S. (2012). Cellular reprogramming: a small molecule perspective. *Current Opinion in Cell Biology*.

[35] Li W., Zhou H., Abujarour R. (2009). Generation of human-induced pluripotent stem cells in the absence of exogenous *Sox2*. *Stem Cells*.

[36] Hawkins R. D., Hon G. C., Lee L. K. (2010). Distinct epigenomic landscapes of pluripotent and lineage-committed human cells. *Cell Stem Cell*.

[37] Shu J., Wu C., Wu Y. (2013). Induction of pluripotency in mouse somatic cells with lineage specifiers. *Cell*.

[38] Lluis F., Ombrato L., Pedone E., Pepe S., Merrill B. J., Cosma M. P. (2011). T-cell factor 3 (Tcf3) deletion increases somatic cell reprogramming by inducing epigenome modifications. *Proceedings of the National Academy of Sciences of the United States of America*.

[39] Zhao R., Daley G. Q. (2008). From fibroblasts to iPS cells: induced pluripotency by defined factors. *Journal of Cellular Biochemistry*.

[40] Niwa H. (2011). Wnt: what's Needed to maintain pluripotency?. *Nature Cell Biology*.

[41] Marson A., Foreman R., Chevalier B. (2008). Wnt signaling promotes reprogramming of somatic cells to pluripotency. *Cell Stem Cell*.

[42] Lyssiotis C. A., Foreman R. K., Staerk J. (2009). Reprogramming of murine fibroblasts to induced pluripotent stem cells with chemical complementation of Klf4. *Proceedings of the National Academy of Sciences of the United States of America*.

[43] Holland J. D., Klaus A., Garratt A. N., Birchmeier W. (2013). Wnt signaling in stem and cancer stem cells. *Current Opinion in Cell Biology*.

[44] Zhao Y., Yin X., Qin H. (2008). Two supporting factors greatly improve the efficiency of human iPSC generation. *Cell Stem Cell*.

[45] Hong H., Takahashi K., Ichisaka T. (2009). Suppression of induced pluripotent stem cell generation by the p53-p21 pathway. *Nature*.

[46] Kawamura T., Suzuki J., Wang Y. V. (2009). Linking the p53 tumour suppressor pathway to somatic cell reprogramming. *Nature*.

[47] Li H., Collado M., Villasante A. (2009). The Ink4/Arf locus is a barrier for iPS cell reprogramming. *Nature*.

[48] Utikal J., Polo J. M., Stadtfeld M. (2009). Immortalization eliminates a roadblock during cellular reprogramming into iPS cells. *Nature*.

[49] Marión R. M., Strati K., Li H. (2009). A p53-mediated DNA damage response limits reprogramming to ensure iPS cell genomic integrity. *Nature*.

[50] Krizhanovsky V., Lowe S. W. (2009). Stem cells: the promises and perils of p53. *Nature*.

[51] Lin T., Chao C., Saito S. (2005). p53 induces differentiation of mouse embryonic stem cells by suppressing Nanog expression. *Nature Cell Biology*.

[52] Shevde N. (2012). Stem cells: flexible friends. *Nature*.

[53] Esteban M. A., Wang T., Qin B. (2010). Vitamin C enhances the generation of mouse and human induced pluripotent stem cells. *Cell Stem Cell*.

[54] Robinton D. A., Daley G. Q. (2012). The promise of induced pluripotent stem cells in research and therapy. *Nature*.

[55] Banito A., Rashid S. T., Acosta J. C. (2009). Senescence impairs successful reprogramming to pluripotent stem cells. *Genes & Development*.

[56] Heiden M. G. V., Cantley L. C., Thompson C. B. (2009). Understanding the warburg effect: the metabolic requirements of cell proliferation. *Science*.

[57] Yoshida Y., Takahashi K., Okita K., Ichisaka T., Yamanaka S. (2009). Hypoxia enhances the generation of induced pluripotent stem cells. *Cell Stem Cell*.

[58] Prigione A., Fauler B., Lurz R., Lehrach H., Adjaye J. (2010). The senescence-related mitochondrial/oxidative stress pathway is repressed in human induced pluripotent stem cells. *Stem Cells*.

[59] Zhu S., Li W., Zhou H. (2010). Reprogramming of human primary somatic cells by OCT4 and chemical compounds. *Cell Stem Cell*.

[60] Li R., Liang J., Ni S. (2010). A mesenchymal-to-epithelial transition initiates and is required for the nuclear reprogramming of mouse fibroblasts. *Cell Stem Cell*.

[61] Samavarchi-Tehrani P., Golipour A., David L. (2010). Functional genomics reveals a BMP-driven mesenchymal-to-epithelial transition in the initiation of somatic cell reprogramming. *Cell Stem Cell*.

[62] Lin T., Ambasudhan R., Yuan X. (2009). A chemical platform for improved induction of human iPSCs. *Nature Methods*.

[63] Ichida J. K., Blanchard J., Lam K. (2009). A small-molecule inhibitor of tgf-*β* signaling replaces *sox2* in reprogramming by inducing *Nanog*. *Cell Stem Cell*.

[64] Yuan X., Wan H., Zhao X., Zhu S., Zhou Q., Ding S. (2011). Brief report: combined chemical treatment enables Oct4-induced reprogramming from mouse embryonic fibroblasts. *Stem Cells*.

[65] Pandian G. N., Sato S., Anandhakumar C. (2014). Identification of a small molecule that turns ON the pluripotency gene circuitry in human fibroblasts. *ACS Chemical Biology*.

[66] Mali P., Chou B.-K., Yen J. (2010). Butyrate greatly enhances derivation of human induced pluripotent stem cells by promoting epigenetic remodeling and the expression of pluripotency-associated genes. *Stem Cells*.

[67] Lee J., Xia Y., Son M.-Y. (2012). A novel small molecule facilitates the reprogramming of human somatic cells into a pluripotent state and supports the maintenance of an undifferentiated state of human pluripotent stem cells. *Angewandte Chemie International Edition*.

[68] Wang Q., Xu X., Li J. (2011). Lithium, an anti-psychotic drug, greatly enhances the generation of induced pluripotent stem cells. *Cell Research*.

[69] Claassen D. A., Desler M. M., Rizzino A. (2009). ROCK inhibition enhances the recovery and growth of cryopreserved human embryonic stem cells and human induced pluripotent stem cells. *Molecular Reproduction and Development*.

[70] Li Z., Rana T. M. (2012). A kinase inhibitor screen identifies small-molecule enhancers of reprogramming and iPS cell generation. *Nature Communications*.

[71] Chen T., Shen L., Yu J. (2011). Rapamycin and other longevity-promoting compounds enhance the generation of mouse induced pluripotent stem cells. *Aging Cell*.

[72] Shi Y., Zou M., Baitei E. Y. (2008). Cannabinoid 2 receptor induction by IL-12 and its potential as a therapeutic target for the treatment of anaplastic thyroid carcinoma. *Cancer Gene Therapy*.

[73] Li Y., Zhang Q., Yin X. (2011). Generation of iPSCs from mouse fibroblasts with a single gene, *Oct4*, and small molecules. *Cell Research*.

[74] Staerk J., Lyssiotis C. A., Medeiro L. A. (2011). Pan-src family kinase inhibitors replace Sox2 during the direct reprogramming of somatic cells. *Angewandte Chemie—International Edition*.

[75] Moon J.-H., Heo J. S., Kim J. S. (2011). Reprogramming fibroblasts into induced pluripotent stem cells with Bmi1. *Cell Research*.

[76] Jung D.-W., Kim W.-H., Williams D. R. (2014). Reprogram or reboot: small molecule approaches for the production of induced pluripotent stem cells and direct cell reprogramming. *ACS Chemical Biology*.

[77] Pasha Z., Haider H. K., Ashraf M. (2011). Efficient non-viral reprogramming of myoblasts to stemness with a single small molecule to generate cardiac progenitor cells. *PLoS ONE*.

[78] Durnaoglu S., Genc S., Genc K. (2011). Patient-specific pluripotent stem cells in neurological diseases. *Stem Cells International*.

[79] Mak L. H., Georgiades S. N., Rosivatz E. (2011). A small molecule mimicking a phosphatidylinositol (4,5)-bisphosphate binding pleckstrin homology domain. *ACS Chemical Biology*.

[80] Ying Q.-L., Wray J., Nichols J. (2008). The ground state of embryonic stem cell self-renewal. *Nature*.

[81] Valamehr B., Robinson M., Abujarour R. (2014). Platform for induction and maintenance of transgene-free hiPSCs resembling ground state pluripotent stem cells. *Stem Cell Reports*.

